# Holographic image generation with a thin-film resonance caused by chalcogenide phase-change material

**DOI:** 10.1038/srep41152

**Published:** 2017-01-24

**Authors:** Seung-Yeol Lee, Yong-Hae Kim, Seong-M. Cho, Gi Heon Kim, Tae-Youb Kim, Hojun Ryu, Han Na Kim, Han Byeol Kang, Chi-Young Hwang, Chi-Sun Hwang

**Affiliations:** 1 Smart I/O Platform Research Department, Electronics and telecommunications research institute, 218 Gajeong-ro, Yuseong-gu, Daejeon 305-700, Korea; 2 Integrated plasmonics and optical device laboratory, School of Electronics Engineering, Kyungpook National University, Daegu, 41566, Korea

## Abstract

The development of digital holography is anticipated for the viewing of 3D images by reconstructing both the amplitude and phase information of the object. Compared to analog holograms written by a laser interference, digital hologram technology has the potential to realize a moving 3D image using a spatial light modulator. However, to ensure a high-resolution 3D image with a large viewing angle, the hologram panel requires a near-wavelength scale pixel pitch with a sufficient large numbers of pixels. In this manuscript, we demonstrate a digital hologram panel based on a chalcogenide phase-change material (PCM) which has a pixel pitch of 1 μm and a panel size of 1.6 × 1.6 cm^2^. A thin film of PCM encapsulated by dielectric layers can be used for the hologram panel by means of excimer laser lithography. By tuning the thicknesses of upper and lower dielectric layers, a color-selective diffraction panel is demonstrated since a thin film resonance caused by dielectric can affect to the absorption and diffraction spectrum of the proposed hologram panel. We also show reflection color of a small active region (1 μm × 4 μm) made by ultra-thin PCM layer can be electrically changed.

In digital holography, spatial light modulators (SLM) have been a key technology in the realization of moving 3D images. One of the most well-known and commercialized approaches to make an SLM is using the liquid-crystal-on-silicon (LCoS) platform^1^,^2^,^3^, as this platform can fully control the 2π phase shift with an electric signal. Although the LCoS platform has been leading the SLM technology due to its competitiveness with regard to visible light control, reducing the size of the active region is limited due to crosstalk of liquid crystals^4^. The smallest pixel pitch for an LCoS-based SLM still remains in about 3.7 μm scale, which is insufficient to provide digital hologram images with an ultimately large viewing angle^5^. It is considered that a pixel pitch of nearly one wavelength or less is required for digital holograms to have a sufficient viewing angle to naturally serve several users without any kinds of eye-tracking techniques^6^,^7^.

With the growth of nanophotonics, many researchers have proposed innovative approaches for overcoming the pixel size limit of digital holography. For example, designing a metasurface, which is an artificial unit cell structure patterned on a thin metallic substrate, has attained the possibility of digital hologram pixels on the subwavelength scale^8^,^9^,^10^,^11^,^12^. Patterned nanorods or nanoslits with different shapes^11^ or orientations^12^ have been used to record the amplitude and phase information of virtual objects. Using metasurfaces can improve digital holography technology by instilling various functionalities, such as extremely high diffraction efficiency^9^, polarization dependent dual-image generation^13^, negative refractions^14^, and the arbitrary phase generation of surface field^15^,^16^. However, active pixel-by-pixel control of phase retardation is quite difficult on a metasurface, as it requires geometrical changes of nanostructure, such as the individual rotation of each nanoslit. Although recent reports demonstrated the active control of metasurfaces, switching of the metasurface was not applied to the unit pixel but instead to the entire metasurface area^17^.

To control light actively, one promising method is to use a phase-change material (PCM). Representative PCMs such as vanadium dioxide (VO_2_) or germanium antimony tellurium alloy (Ge_2_Sb_2_Te_5_, GST) have been thoroughly researched for integrated active optical devices. For example, VO_2_ undergoes a thermally driven phase change near 68 °C, transforming from the semiconductor to the metallic phase state. With VO_2_, active optical devices such as perfect light absorbers^18^, switchable nanoantennas^19^,^20^, and ring resonators^21^ have been reported, but devices based on VO_2_ have drawbacks as they have to maintain the temperature in certain region to retain its own phase state.

On the other hand, the GST alloy is a phase-change material which has been conventionally used in optical data storage devices such as DVDs^22^. It is known that the heating of the GST layer to 150 °C crystallizes the material, whereas total melting and quenching of GST at 600 °C can return the material to an amorphous state^23^. Between amorphous and crystalline states, significant changes in the refractive index and extinction coefficients which reach nearly ~0.5 at visible frequencies have been reported^24^. Moreover, the phase change of GST film is non-volatile, repetitive, and easily controlled by both electric and photonic stimuli^25^. Therefore, it has been reported that only a few nanometers of GST film can result in a total change of the reflected color, with the inserting of the GST film into the dielectric cavity^24^. Due to these benefits, GST has not only been used in next-generation non-volatile memory devices^25^,^26^, but also in upcoming subwavelength-scale display pixels^24^. Periodic nano-disks patterned on a metal/GST composite layers has been reported for tunable perfect absorbing metamaterials in the visible and mid-infrared ranges^27^,^28^, and optically reconfigurable metasurfaces based on GST were very recently reported^29^. However, there are no reports on the full implementation of a digital hologram which can be directly observed by the human eye with a phase-changeable chalcogenide compound.

The presented report demonstrates that an ultra-thin GST layer inserted into a dielectric medium can enhance the diffraction efficiency and can be used for color-selective diffraction. The thicknesses of the dielectric layers play a significant role in the high diffraction efficiency, as the diffraction-enhanced wavelength is mainly determined by thin-film resonance condition of the dielectric cavity. It is shown that the resonance condition after the insertion of GST film can be red-shifted or blue-shifted from the original resonance condition according to the material state of GST film, allowing a large phase change of the reflection coefficient to be achieved before and after the crystallization of the GST. Computer-generated hologram (CGH) patterns with a 1 μm (or 2 μm) pixel pitch and a 16k × 16k resolution are shaped by the local phase change of GST film, which was accomplished by excimer laser lithography. An additional experiment for electrical switching of a small active region (1 μm × 4 μm) is done for showing the potential of proposed scheme as an electrically-driven SLM.

## Materials and Methods

### Fabrication of the hologram panel with laser lithography

**Figure 1:**
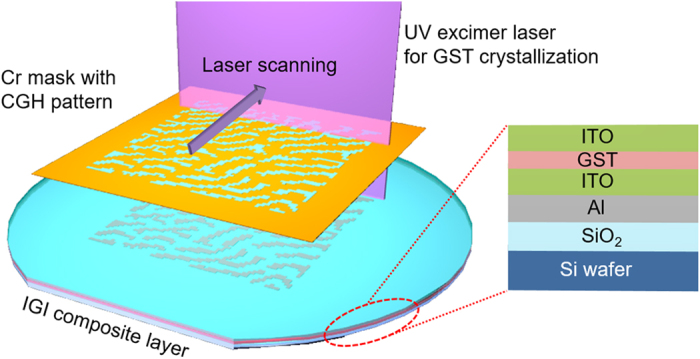
Schematic of the proposed multi-layered GST-based sample and its hologram recording process.

The basic scheme of the proposed thin-film GST-based digital hologram is shown in Fig. 1. Initially, 300 nm SiO_2_ film is deposited as a thermal isolation layer onto a six-inch Si wafer by plasma-enhanced chemical vapor deposition (PECVD). Next, a 300 nm Al layer is sputtered for reflection layer. Dielectric-GST-dielectric composite layers are then sputtered onto the metal surface in a step by step manner. Here, we use indium-tin-oxide (ITO) for the dielectric layer owing to its high conductivity and heat dissipation rate. The initially sputtered GST film is in an amorphous state. The thicknesses of ITO layers vary in this study so that color selectivity can be realized. A Cr mask with a CGH pattern carved on quartz substrate was also prepared for the local phase change of the GST film. After attaching the mask to the fabricated ITO-GST-ITO (IGI) composite layer, an excimer laser with a wavelength of 308 nm, a pulse width of 30 ns, a repetition rate of 600 Hz, and a maximum pulse energy of 580 mJ/cm^2^ is used for the local crystallization of the GST film. The excimer laser has a line-shaped beam profile with 400 μm width parallel to the scanning direction, and more than six inches of the width perpendicular to the scanning direction, as illustrated in Fig. 1. By varying the pulse energy illuminated on the sample, we found that the appropriate energy region for crystallizing the GST film without damaging the other layers such as Al and ITO, is 60–80 mJ/cm^2^ (See supplementary part A for details).

### Material characteristics of the GST film

**Figure 2:**
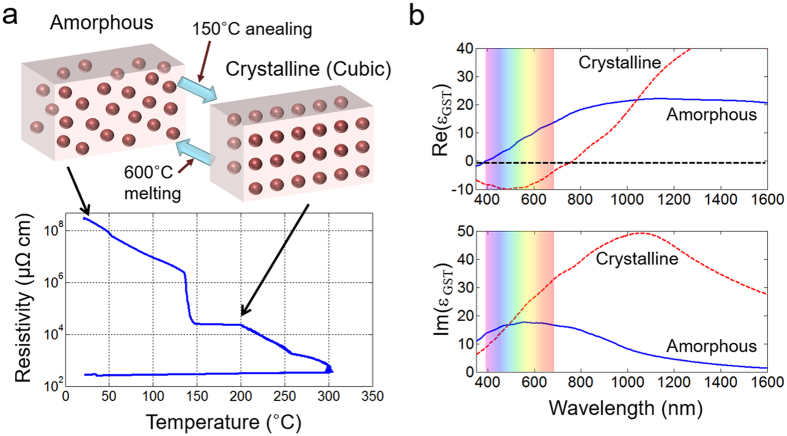
(**a**) Phase-change diagram of GST alloy by measuring its electrical resistivity in a temperature-controlled chamber. (**b**) Optical characteristics of the 20-nm GST alloy in the amorphous (blue solid) and crystalline (red dashed) states using an ellipsometer system.

As reported in many previous works using the GST alloy^23^,^24^,^25^,^26^,^27^,^28^, GST undergoes a phase change which can be repetitively switched. Because the material characteristics of GST can differ slightly when different composition ratios and sputtering conditions are used^23^, it was necessary to measure the electrical resistances and optical constants of the actual GST alloy used in this work for amorphous and crystalline states. In Fig. 2a, the electric resistivity of the GST while varying the temperature is shown. A 20-nm-thick GST film sputtered on a SiO_2_ substrate served as the monitoring sample, where the measurement of the resistivity was done inside a furnace chamber under an N_2_ gas flowing condition. The initially sputtered GST film had an amorphous state with very high resistivity, and crystallization occurred when the film was heated to 150 °C. Therefore, in accordance with the increase of temperature, the resistivity of the GST film is reduced and an abrupt change occurs near 150 °C, which is a phase change from the amorphous to the crystalline (cubic) state. After reaching 300 °C, cooling of the GST film does not restore its high resistivity; i. e., the low resistivity condition is maintained. Such a non-volatile phase change characteristic of GST has been numerously applied to develop high-density phase-change memory (PRAM) devices^30^ as well as classical optical memory such as DVDs.

Otherwise, the optical permittivity of the thin GST film before and after crystallization is our main concern for optical modulation. Figure 2b shows real (Re(ε_GST_)) and imaginary (Im(ε_GST_)) parts of GST permittivity levels as measured by an ellipsometer system (EC-400, J. A. Woollam Co.). In this study, the region of interest is the visible light range at wavelengths of 400 nm to 700 nm. In this range, it is noteworthy that Re(ε_GST_) has a positive value in an amorphous state, whereas a negative value is noted in a crystalline state. However, we cannot clearly classify these states as dielectric or metallic phases due to the high imaginary part of ε_GST_ for both phases, as depicted in the lower plot of Fig. 2b. Nevertheless, the sign-change of Re(ε_GST_) plays a crucial role to the shifting-direction of absorption resonance inside the IGI composite layer, which will finally enhance the diffraction efficiency of the proposed hologram image. The details of the theoretical analysis will be given in the design rule section.

The optical constants of ITO that are used in the simulation are also measured by the same ellipsometer system. (See supplementary Part B) The permittivity of other materials such as Al and SiO_2_ was sourced from the literature^31^.

### CGH pattern generation

The CGH pattern carved on the Cr mask was calculated using the method introduced by K. Matsushima^32^. The number of hologram pixels is 16,384 × 16,384, and two types of pixel pitches, 1 μm and 2 μm, are designed. To improve the memory issues for calculation, the CGH patterns are segmented into 8 × 8 blocks with a size of 2,048 × 2,048 pixels. To observe the holographic image with different depth information, two distanced image planes of *z*_1_* = *15 cm and *z*_2_* = *12.5 cm are applied for the 1 μm pixel pitch CGH pattern. Here, we define the location of the CGH pattern as *z*_CGH_* = *0 cm. The CGH pattern is designed to reconstruct a checkerboard image at *z*_1_, and letters ‘NANO SLM’ at *z*_2_. For the case of 2 μm pixel pitch CGH mask, *z*_1_* = *60 cm and *z*_2_* = *50 cm are applied. The total size of hologram image is almost same as that of CGH panel, which are about 1.5 cm, 3 cm for 1 μm, 2 μm pixel pitch, respectively. Therefore, our hologram panel can be clearly observed by human eye without any optical instruments.

## Results and Discussion

### Design rule of IGI composite layers

**Figure 3:**
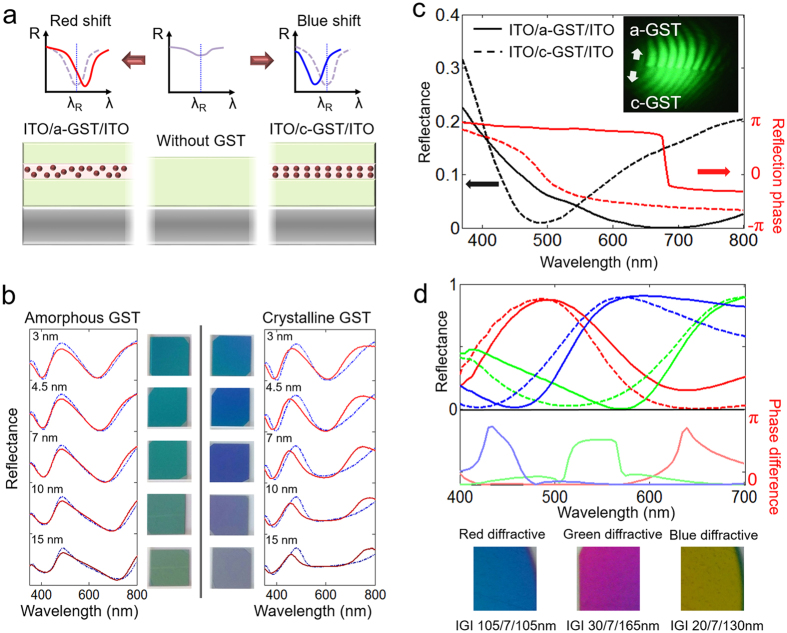
(**a**) Schematic diagram for illustrating the major variation of the optical spectrum when amorphous (or crystalline) thin GST film is inserted into ITO on a metal substrate. (**b**) Simulation (blue dash-dotted) and experimental (red solid) results of the reflection spectra to observe the effects of the GST thickness. (**c**) Reflectance and reflection phase of the IGI composite layer designed for broadband visible light diffraction (IGI 30/7/30 nm). The upper-right inset shows the abrupt phase difference of reflected light as measured by a Michelson interferometer experiment. (**d**) The upper-side plots show the experimental results of the reflection spectra for amorphous (solid lines) and crystalline (dashed lines) GST inserted panels designed for red, green, and blue color-selective diffraction. The lower-side plots show the numerical results of the reflection phase difference 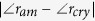 between the amorphous and crystalline GST insertion cases. The bottom images show the color of reflection panels and their IGI thickness conditions.

In this section, we would like to discuss how to design a hologram image with the phase change of ultra-thin GST film. In order to generate a hologram image, light passing through the CGH panel should be efficiently diffracted and these diffracted lights form a hologram image at the image plane. Therefore the brightness of the reconstructed hologram image is affected by the first-order diffracted light from the CGH image^6^, and it is necessary to optimize the diffraction efficiency of the gratings formed by the GST phase transition. Since our scheme is a binary hologram, which has the first-order diffraction efficiency (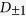) with an approximated form of 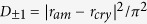 33, where *r*_*am*_ (or *r*_*cry*_) is the reflection coefficients of the IGI composite layer when the intermediate GST film is amorphous (or crystalline) phase state, respectively. To maximize the diffraction efficiency, we choose a strategy to increase the phase difference between *r*_*am*_ and *r*_*cry*_ close to π. Figure 3a shows the aspect of reflection spectrum of our IGI composite layer when inserted GST film is phase-changed. Without considering the thin GST layer, the air-ITO-Al layer has its own destructive thin-film interference resonance condition at 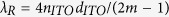, where *m, n*_*ITO*_ and *d*_*ITO*_ are the natural number for the cavity resonance order, the refractive index of ITO, and the thickness of ITO, respectively. When GST film is inserted into the ITO layer, two major changes appear in the spectral response. First, the absorption resonance dip in the destructive thin-film interference resonance condition is enlarged due to the high lossy characteristic of the GST film. Second, interestingly, the absorption resonance dip is red-shifted after the insertion of the amorphous GST film, whereas it is blue-shifted after insertion of the crystalline GST film.

Such characteristics are plotted in Fig. 3b in both transfer matrix method (TMM) numerical simulations and experimental measurements. As discussed in the material characteristic section, such different directionalities of the resonance shift can be explained by the sign-change of real-part permittivity of GST. The sign-change of real permittivity can increase or decrease the effective cavity length formed by IGI composite layer. More precisely, we theoretically confirmed that the resonance dip is red-shifted when 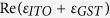 is positive, whereas it is blue-shifted when 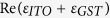 is negative (See supplementary part C). Moreover, from Fig. 3b, we can find the appropriate thickness region of the GST layer for the resonance shift phenomenon. A GST layer (3 nm, 4.5 nm) which is too thin cannot significantly shift the resonance wavelength between the amorphous and crystalline phase change; therefore, the reflected color (and reflection phase difference) does not change sufficiently. On the other hand, the direct reflection at the upper ITO-GST interface is increased when too thick GST layer (15 nm, 20 nm) is used, which makes the reflected color from the IGI composite layer into gray tone for both material phase states. The appropriate thickness of the GST layer has been determined to be close to 7 nm, which also showed the most significant color change in a previous study^24^. We also numerically confirmed in our recent work that the diffraction efficiency has the highest value when this GST thickness condition is used as well as color difference^34^.

When an absorption resonance dip is located at a certain wavelength, it is known that an abrupt phase change should appear at the resonance wavelength owing to the Kramers–Kronig relation. Figure 3c shows the simulation results of the reflectance and reflection phase of an IGI composite layer under the 30/7/30 nm condition, which is modeled as a broadband diffraction condition. In this case, *d*_*ITO*_ is only 60 nm; therefore, the first-order resonance (*m* = 1), which has fairly broad bandwidth, appears in the visible light spectrum. The resonance dips are located at 680 nm for the amorphous GST and 480 nm for the crystalline GST. It is shown that the reflection phase abruptly changes at the resonance wavelengths in each case. Therefore phase difference (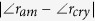) is sufficiently large in the range of 480 nm to 680 nm, which contains nearly the entire range of the visible-light spectrum. The inset of Fig. 3c shows the experimental results to verify the abrupt phase change between amorphous and crystalline IGI composite film using the Michelson interferometer system at 532 nm. The lower-half of the sample was changed to the crystalline state using excimer laser prior to the interference experiment. This figure clearly shows that an abrupt phase change occurs before and after the crystallization of the GST layer, which will be quite useful for diffracting light and generating a binary hologram.

If we use a thicker ITO layer, the cavity length of thin-film resonator is increased. Therefore, first-order resonance spectrum moves to the infrared wavelength region, and higher order resonances appears in the visible wavelength. Because these higher order resonances have much narrower spectra, we can use these narrower resonance to design a color-selective diffraction grating by simply tuning the thickness of the ITO and the relative position of the GST layer. In Fig 3d, we show the reflected power and phase spectra for three different thickness compositions of the IGI layer for red, green, blue color-selective diffraction. Similar to the plot shown in Fig 3c, the regions for large phase differences are shown for each color by the GST phase change, but these regions are only applied to the specified RGB color region in each case. In our configuration, the wavelength region for the high-diffraction efficiency condition is matched to that for the absorption resonance, where the direct reflection is minimized. Therefore, the diffraction colors of IGI composite lasers are opposite to their own color. For example, the red-selective diffraction panel is cyan in color, the green-selective one is magenta, and the blue-selective one is yellow-gold, as shown in the bottom images of Fig. 3d. Such a complementary color panel may increase the viewing contrast of hologram images when the reflection panel is directly observed by the human eye. For the broadband diffraction case, the absorption resonance is broadly positioned along the visible light spectrum. Therefore, the color of panel seems to dark-blue for the amorphous state and brown for the crystalline state, respectively.

### Reconstructed hologram image

**Figure 4:**
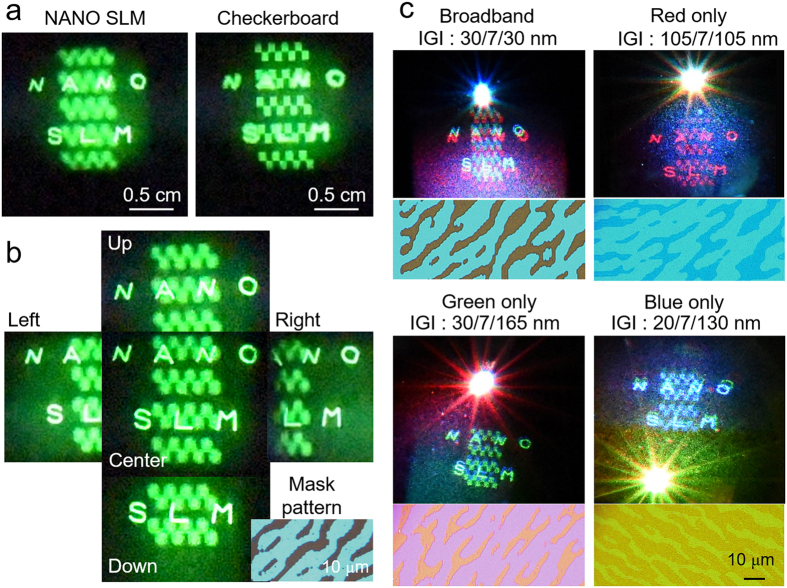
Reconstructed hologram images from the proposed 1 μm pixel pitch GST-based CGH patterns to verify (**a**) the different depth information and (**b**) the full parallax characteristics. (**c**) Reconstructed hologram images to verify the color-selective diffraction panels. The Lower-side image of each hologram image shows a microscope view of each CGH pattern. (2 μm pixel pitch).

**Figure 5:**
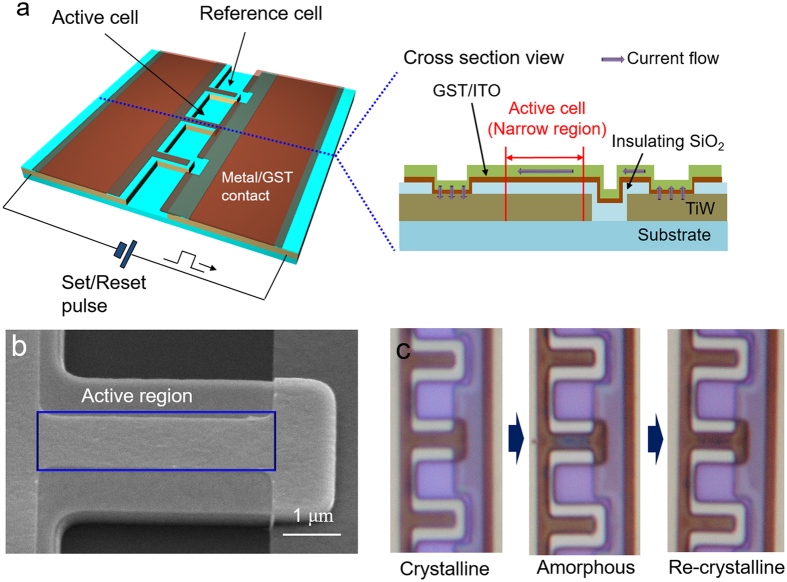
(**a**) Fabricated structure for phase-changing of thin GST film with electric pulse. (**b**) SEM image of the active region that used in electric pulse experiment. (**c**) Microscope views of the active region and reference cells before and after the GST phase transition.

The reconstructed hologram images from the CGH patterns with a pixel pitch 1 μm and with the 30/7/30 nm IGI thickness condition are shown in Fig. 4a. A Green laser diode source (Ventus 532) of 15 mW at 532 nm is used in the experiments shown in Fig. 4a,b, which is also consistently used in interferometer experiment in Fig. 3c and the green source of color-selective experiment in Fig. 5c. To ensure the depth difference between the two different focal planes, we captured two images of which the camera focus on letters ‘NANO SLM’ and checkerboard respectively. It is clearly shown that the two images are located on different focal planes. In Fig. 4b, we also captured side-view images of the reconstructed hologram to show the full-parallax characteristics. Each image is approximately 6° away from the center image. These images clearly show that the relative viewing position between the checkerboard and the letters are changed according to the tilting direction. Note that the checkerboard image is in front of the letters in our case; therefore, the letters seems to move in the tilting direction with respect to the checkboard image. Here, it is noteworthy that the maximum viewing angle of our system is restricted by the distance between CGH panel and image plane (calculated by 6.1°), not the theoretical limit of diffractive angle of 1 um pixel pitch at 532 nm (See supplementary Part D). Moreover, additional numerical simulations show that the diffraction efficiency is almost linearly decreased when the portion of active area compared to non-active region is reduced. Details can be found in supplementary Part E.

Next, in Fig. 4c, the reconstructed hologram from the color-selective diffraction panel with the 2 μm pixel pitch is shown. Here, we used three different laser diodes of red, green, and blue, at 660 nm, 532 nm, and 473 nm, respectively. The power levels of each laser source are fixed at 15, 15, and 25 mW for the red, green, and blue lasers, respectively, because the diffraction of blue light was slightly weaker for all hologram panels. To show the color-selective diffraction characteristics clearly, the RGB laser sources simultaneously illuminate the sample, and we capture both the diffracted hologram image and non-diffracted reflection beam. The bright spot near the hologram panel shows non-diffracted beam, clearly demonstrating that only the designed color is diffracted from each hologram panel. The broadband-diffraction panel shows the hologram image with RGB simultaneously, whereas the red-diffraction panel shows almost perfect color selectivity. Although green-diffraction (blue-diffraction) panel shows non-filtered blue (green) diffracted light weakly, the dominant diffraction color can still be clearly observed. As shown in Fig. 3d, the designed blue-diffractive panel show better performance at blue wavelength shorter than 473 nm. Therefore, we expect that a better color-filtered image can be achieved if we replace the blue laser wavelength to shorter range or improve the spectrum of the IGI composite layer to be sharper by designing the ITO thickness more precisely. The lower-side images in Fig. 4c are microscope images of the CGH pattern of each corresponding hologram panel.

### Additional experiment: Phase change of ultra-thin GST with electric pulse

One of the promising characteristic of GST is that the phase change phenomenon can also be achieved by electric pulse signal. In this section, we demonstrate that a small region (1 μm × 4 μm) of 7 nm GST film can also be phase-changed by electric pulse. A schematic of fabricated structure is shown in Fig. 5a. In the right-side of Fig. 5a, cross-section view of the fabricated sample is shown. Currents are laterally flow through the upper ITO and Joule heats generated from the upper ITO can increase the temperature of GST, which is a principle of the phase change of active region.

Here, we used three steps of photo lithography on a SiO_2_-coated Si wafer in the fabrication process. The first photomask is used for the patterning of a 200 nm TiW metal layer which serves as the electrodes as well as the reflection pad of active region. We chose TiW instead of Al for uniformity of the film and for fabrication stability during the harsh ITO/GST etching process. The second photomask is used for the patterning of 50 nm of SiO_2_. In this experiment, lower dielectric must be an electrical and thermal insulator to focus the lateral current flow within the upper ITO of active region. Therefore we choose SiO_2_ layer for lower dielectric instead of ITO. Since the SiO_2_ layer may replaces the lower ITO used in the main experiment, thickness of SiO_2_ is controlled to have the similar optical path length with the lower ITO in the main experiment. Hence, to mimic a spectrum of broadband diffractive panel, thickness of SiO_2_ should be 50 nm to replace the 30 nm ITO layer considering the refractive index differences of these materials. (n_ITO_ = 2.1, n_SiO2_ = 1.4). The third photomask is used for patterning the upper ITO/GST layer. The upper ITO also have a role for protecting the GST region during the harsh etching process as well as path for lateral current flow. The overall GST region became crystalline state during the ITO/GST etching process, as the temperature increased up to 180 °C during this process. Therefore, the etched GST is pre-crystallized, unlike the un-patterned film used in laser lithography experiments.

In Fig. 5b, a SEM image of the device used in our electric pulse experiment is shown. The region where the GST phase transition occurs is a narrow rectangular region of 1 μm × 4 μm in size, as marked by the blue line. The microscope images before and after the phase transitions are shown in Fig. 5c. Two reference cells with an identical layered-structure but which are electrically opened, are shown near the active cell to compare the change of color. Due to the narrow width of the active region, the laterally flowing currents converge and the temperature may increase to the greatest extent at the active region. The temperature threshold of the GST melting point can only be achieved in the active region; therefore, a local GST phase change can be achieved.

Since the GST film is pre-crystallized in this experiment, a crystalline-to-amorphous phase transition initially occurs. To make the GST amorphous, rapid melting at 600 °C and rapid quenching are needed. Therefore, we applied high voltage and, a short pulse width set signal of 9 V for 500 ns. The middle image in Fig. 5c shows the sample after the set pulse is applied, clearly showing that the color of the active cell region changes from brown to dark blue. It should be noted that this color change is in good agreement with the theoretically calculated spectrum of the active cell region (ITO/GST/SiO_2_/TIW of 30/7/50/200 nm) (See supplement part F). This condition is designed to have a similar resonance spectrum with the broadband color diffraction condition of the laser lithography experiment (IGI: 30/7/30 nm) except for the slightly lower reflectance due to the use of TiW instead of Al. By comparing Fig. 3c and supplementary Part F, the degradation of the average reflectance caused by using TiW instead of Al in visible light range is found to be less than 0.05.

Next, it is noteworthy to show the amorphous-to-crystalline phase transition by applying a reset pulse signal. Because the crystalline form of GST requires relatively slow heating at a moderate temperature of 150 °C, a reset pulse signal of 5 V for 100 μs is used. The right-side image in Fig. 5c show that the color of the active cell region turn back to brown. The slight difference between the initial brown color and the re-crystallized brown color originates from the different methods of crystallization. In supplementary Part G, we also conduct the similar experiment with different ITO/GST/SiO_2_ thickness condition, which is designed to have bluish color at amorphous and crystalline state respectively. As expected, this thickness condition is related to red-selective diffractive panel. Experimental results for smaller active region are also included.

## Conclusion

In conclusion, our results reveal that a phase transition of ultra-thin GST film inserted into a dielectric layers can be used to realize a color-selective hologram panel. The proposed panel uses the dramatic optical permittivity change of GST during the phase transition, which can shift the resonance condition of thin film interference in the opposite direction before and after GST crystallization. The resonance condition of thin film interference can be directly applied to design diffracting color spectra, resulting in a broadband, red, green, and blue color diffracting hologram panel. We expect that such a characteristic may be applied to realize a full-color digital hologram from a single panel without the use of any types of additional color filters. The additional experiment shows that the proposed dielectric-embedded thin-GST film can also be switched by electric signal as well as optical signal. We expect that such flexibility in switching methods is quite useful for developing a novel type of SLM in the near future.

## Additional Information

**How to cite this article**: Lee, S.-Y. *et al*. Holographic image generation with a thin-film resonance caused by chalcogenide phase-change material. *Sci. Rep.* **7**, 41152; doi: 10.1038/srep41152 (2017).

**Publisher's note:** Springer Nature remains neutral with regard to jurisdictional claims in published maps and institutional affiliations.

## Supplementary Material

Supplementary Material

## References

[b1] Konforti N., Marom E. & Wu S. T. Phase-only modulation with twisted nematic liquid-crystal spatial light modulators. Optics Letters 13, 251–253 (1988). 19742044 10.1364/ol.13.000251

[b2] McKnight D. J., Johnson K. M. & Serati R. A. 256 × 256 liquid-crystal-on-silicon spatial light modulator. Applied Optics 33, 2775–2784 (1994). 20885636 10.1364/AO.33.002775

[b3] Hu L. . Phase-only liquid-crystal spatial light modulator for wave-front correction with high precision. Optics Express 12, 6403–6409 (2004). 19488289 10.1364/opex.12.006403

[b4] Lueder E. Liquid Crystal Displays: Addressing Schemes and Electro-Optical Effects. Wiley, 2010.

[b5] HoloEye Photonics A. G.. GAEA: 10 Megapixel Phase Only Spatial Light Modulator. 2015.

[b6] Schnars U. & Jueptner W. Digital holography. Springer, 2005.

[b7] Reichelt S. . Holographic 3-D Displays - Electro-holography within the Grasp of Commercialization. In: Costa N., Cartaxo A. Advances in Lasers and Electro Optics. InTech, 2010.

[b8] Ni X., Kildishev A. V. & Shalaev V. M. Metasurface holograms for visible light. Nature communications 4, doi: 10.1038/ncomms3807 (2013).

[b9] Zheng G. . Metasurface holograms reaching 80% efficiency. Nature nanotechnology 10, 308–312 (2015). 10.1038/nnano.2015.2 25705870

[b10] Wen D. . Helicity multiplexed broadband metasurface holograms. Nature communications 6, doi: 10.1038/ncomms9241 (2015). PMC4579785 26354497

[b11] Huang L. . Three-dimensional optical holography using a plasmonic metasurface. Nature communications 4, doi: 10.1038/ncomms3808 (2013).

[b12] Kildishev A. V., Boltasseva A. & Shalaev V. M. Planar Photonics with Metasurfaces. Science 339, doi: 10.1126/science.1232009 (2013). 23493714

[b13] Chen W. T. . High-Efficiency Broadband Meta-Hologram with Polarization-Controlled Dual Images. Nano Letters 14, 225–230 (2014). 24329425 10.1021/nl403811d

[b14] Fleury R., Sounas D. L. & Alù A. Negative Refraction and Planar Focusing Based on Parity-Time Symmetric Metasurfaces. Physical Review Letters 113, 023903 (2014). 25062184 10.1103/PhysRevLett.113.023903

[b15] Lee S. Y., Kim K., Lee G. Y. & Lee B. Polarization-multiplexed plasmonic phase generation with distributed nanoslits. Optics Express 23, 15598–15607 (2015). 26193539 10.1364/OE.23.015598

[b16] Song E. Y. . A double-lined metasurface for plasmonic complex-field generation. Laser and Photonics Reviews 10, 299–308 (2016).

[b17] Zheludev N. I. & Kivshar Y. S. From metamaterials to metadevices. Nature Materials 11, 917–924 (2012). 23089997 10.1038/nmat3431

[b18] Kats M. A. . Ultra-thin perfect absorber employing a tunable phase change material. Applied Physics Letters 101, 221101 (2012).

[b19] Seo M. . Active terahertz nanoantennas based on VO_2_ phase transition. Nano Letters 10, 2064–2068 (2010). 20469898 10.1021/nl1002153

[b20] Park J. B. . Tunable subwavelength hot spot of dipole nanostructure based on VO_2_ phase transition, Optics Express 21, 15205–15213 (2013). 23842306 10.1364/OE.21.015205

[b21] Appavoo K. & Haglund R. F. Jr Detecting nanoscale size dependence in VO_2_ phase transition using a split-ring resonator metamaterial. Nano Letters 11, 1025–1031 (2011). 21306154 10.1021/nl103842v

[b22] Sun Z., Zhou J. & Ahuja R. Structure of phase change materials for data storage. Physical Review Letters 96, 055507 (2006). 16486951 10.1103/PhysRevLett.96.055507

[b23] Kolobov A. V. . Understanding the phase-change mechanism of rewritable optical media. Nature Material 3, 703–708 (2004). 10.1038/nmat1215 15359344

[b24] Hosseini P., Wright C. D. & Bhaskaran H. An optoelectronic framework enabled by low-dimensional phase-change film. Nature 511, 206–211 (2014). 25008527 10.1038/nature13487

[b25] Wang W. J. . Nonvolatile phase change memory nanocell fabrication by femtosecond laser writing assisted with near-field optical microscopy. Journal of Applied Physics 98, 124313 (2005).

[b26] Ríos C. . Integrated all-photonic non-volatile multi-level memory. Nature Photonics 9, 725–733 (2015).

[b27] Tittl A. . A switchable mid-infrared plasmonic perfect absorber with multispectral thermal imaging capability. Advanced Material 27, 4597–4603 (2015). 10.1002/adma.201502023 26173394

[b28] Cao T. . Broadband polarization-independent perfect absorber using a phase-change metamaterial at visible frequencies. Scientific Reports 4, doi: 10.1038/srep03955 (2014). PMC3912474 24492415

[b29] Wang Q. . Optically reconfigurable metasurfaces and photonic devices based on phase change materials. Nature Photonics 10, 60–65 (2016).

[b30] Yoon S. M. . Sb–Se based phase-change memory device with lower power and higher speed operations. IEEE Electron Device Letters 27, 445–447 (2006).

[b31] Palik E. D. Handbook of Optical Constants of Solids. Academic Press, 1991.

[b32] Matsushima K. & Nakahara S. Extremely high-definition full-parallax computer-generated hologram created by the polygon-based method. Applied Optics 48, H54–H63 (2009). 19956302 10.1364/AO.48.000H54

[b33] Benton S. A. & Bove V. M. Jr. Holographic imaging. John Wiley & Sons, 2008.

[b34] Lee S. Y. . Design method of tunable pixel with phase-change material for diffractive optical elements. ETRI Journal (in review).

