# Supplementary material for: Holographic image generation with a thin-film resonance caused by chalcogenide phase-change material
Source: Sci Rep. 2017 Jan 24;7:41152. doi: 10.1038/srep41152 (PMC5259724; doi:10.1038/srep41152)
Supplement: Supplementary Material [file srep41152-s1.pdf]

Supplementary Material to the manuscript "Holographic image generation with a thin-film resonance caused by chalcogenide phase-change material"

Seung-Yeol Lee*, Yong-Hae Kim, Seong-M. Cho, Gi Heon Kim, Tae-Youb Kim, Hojun Ryu, Han Na Kim, Han Byeol Kang, Chi-Young Hwang, and Chi-Sun Hwang

Smart I/O Platform Research Department, Electronics and telecommunications research institute, 218 Gajeong-ro, Yuseong-gu, Daejeon 305-700, Korea.

Corresponding author e-mail: *sylee87snu@gmail.com

Phone number: +82-53-950-6608

Part A. Optimization of the laser exposure conditions for the GST phase change

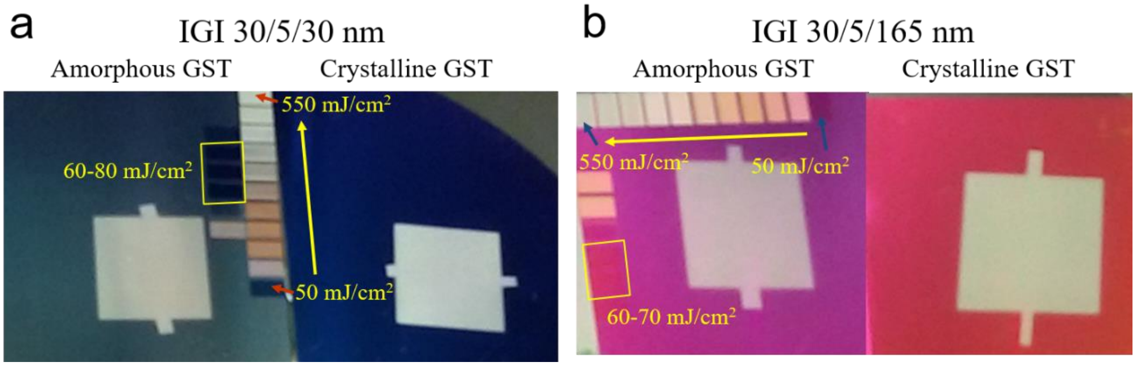

Figure S1. Excimer laser exposure test for the crystallization of the GST layer of the IGI panel. Various laser exposure power levels ranging from 50 mJ/cm^2 to 550 mJ/cm^2 are tested for (a) the IGI: 30/5/30 nm and (b) the IGI: 30/5/165 nm composite layer deposited on the 300 nm Al layer.

Figure S1 shows the experimental results attempting to find appropriate pulse energy range for the crystallization of GST without damaging the GST film or the other layers. The left-side wafer sample shown in Fig. S1(a) shows the IGI composite layer of 30/5/30 nm on Al, which has amorphous GST in its initial state. On the other hand, the image on the right-side is that of the sample for which the GST is fully

crystallized over the entire region after annealing the sample in a furnace at 200 °C for three hours. The square spots shown in the image of the amorphous GST sample are the region where the excimer laser source directly illuminates the sample at various pulse power levels. The left-side array of squares shows the rough tuning experiment conducted from 50 to 550 mJ/cm^2 (down to up) with an increment of 50 mJ/cm^2, whereas the right-side squares show the fine tuning experiment from 30 to 100 mJ/cm^2 with an increment of 10 mJ/cm^2 (no color change for the 30 and 40 mJ/cm^2 cases). The appropriate pulse power region for GST crystallization was found to be 60-80 mJ/cm^2 in this experiment by comparing the changed panel color with that of the fully crystallized sample. A similar experiment was also done for the other IGI thickness conditions of 30/5/165 nm, as shown in Fig. S1(b). The large square spot shown at the center of both samples is the region where GST is not deposited (only ITO layers are deposited).

Part B. Experimentally measured refractive index of ITO

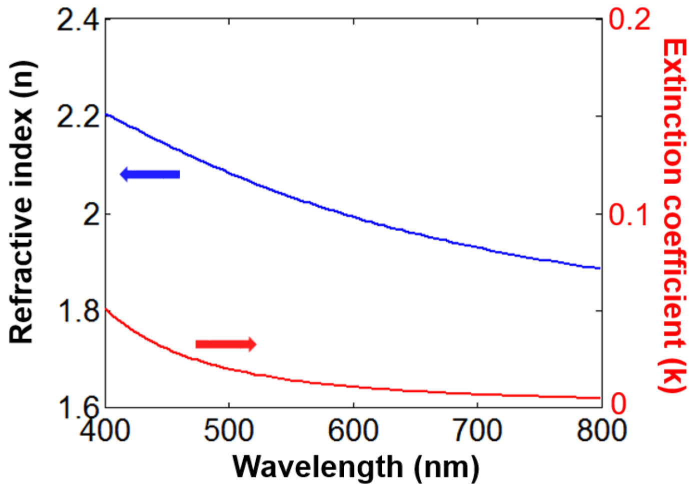

Figure S2. Experimentally measured refractive index of ITO layer

In Fig. S2, the refractive index of ITO experimentally measured by the ellipsometer system used in the simulation is shown.

Part C. Theoretical analysis of the reflection from the IGI-metal composite layer

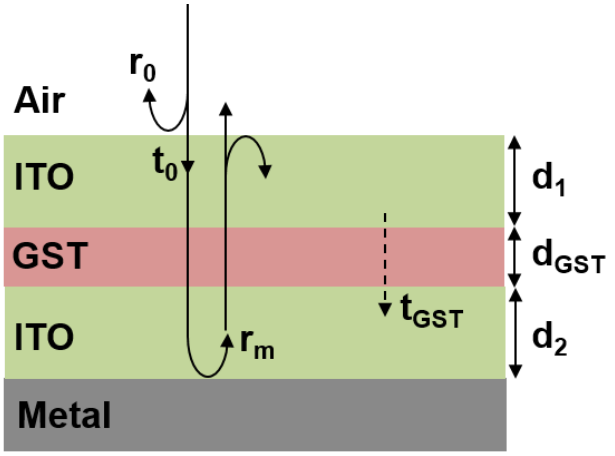

Figure S3. Schematic for explaining the reflection characteristics of the IGI-metal composite layer.

The reflectance from the proposed IGI composite layer can be simply solved by numerical methods such as the transfer matrix method (TMM). However, we would like to apply a simplified model to gain physical insight into the resonance shift phenomenon caused by the insertion of the GST layer. Here, we assume that the thickness of the GST layer (d_{GST}) is sufficiently thin so that the reflection at the ITO-GST interface could be disregarded. Then, the overall reflection coefficient of the composite layer (r_{com}) can be expressed as,

$$r_{com} = r_0 + \frac{t_0^{\,2} r_m P_{IGI}^{\;2}}{1 + r_0 r_m P_{IGI}^{\;2}}, \qquad \text{(S1)}$$

where r_0, t_0, and r_m indicate the reflection coefficient at the air-ITO interface, the transmission coefficient at the air-ITO interface, and the reflection coefficient at the ITO-metal interface, respectively. P_{IGI} is a propagation coefficient through the ITO-GST-ITO layer. It can be approximated to,

$$P_{IGI} = t_{GST} \exp(j\sqrt{\varepsilon_{ITO}}\, k_0 (d_1 + d_2)). \qquad \text{(S2)}$$

Note that we ignore the multiple reflection caused by the reflection at the ITO-GST interface due to the sufficiently thin GST. The wavelength of the thin-film resonance can be controlled by the phase propagation coefficient P_{IGI}, which is mainly governed by the sum of the ITO thicknesses, but which can also be altered by the phase of t_{GST}. The coefficient t_{GST} can be expressed as,

$$t_{GST} = \frac{4\sqrt{\varepsilon_{ITO}\varepsilon_{GST}}}{\left(\sqrt{\varepsilon_{ITO}} + \sqrt{\varepsilon_{GST}}\right)^2 P_{GST}^{\;-1} - \left(\sqrt{\varepsilon_{ITO}} - \sqrt{\varepsilon_{GST}}\right)^2 P_{GST}}. \qquad \text{(S3)}$$

Here, P_{GST} is the propagation coefficient through the thin GST film, which is expressed as $P_{GST} = \exp(j\sqrt{\varepsilon_{GST}}\, k_0 d_{GST})$. By submitting P_{GST}, Equation (S3) can be written as,

$$t_{GST} = \frac{2\sqrt{\varepsilon_{ITO}\varepsilon_{GST}}}{2\sqrt{\varepsilon_{ITO}\varepsilon_{GST}}\cos(\sqrt{\varepsilon_{GST}}\, k_0 d_{GST}) - j\left(\varepsilon_{ITO} + \varepsilon_{GST}\right)\sin(\sqrt{\varepsilon_{GST}}\, k_0 d_{GST})}. \qquad \text{(S4)}$$

Note that $t_{GST}=1$ when $d_{GST}=0$. Equation (S4) can be approximated by a first-order Taylor series with a very small d_{GST} value, which can be expressed as,

$$t_{GST}\approx\left(\frac{1}{1-i\left((\varepsilon_{ITO}+\varepsilon_{GST})/2\sqrt{\varepsilon_{ITO}}\right)k_0d_{GST}}\right)\approx(1+i\left((\varepsilon_{ITO}+\varepsilon_{GST})/2n_{ITO}\right)k_0d_{GST})\,. \qquad \text{(S5)}$$

Equation (S5) implies that t_{GST} has phase-lead characteristics when $\mathrm{Re}(\varepsilon_{ITO}+\varepsilon_{GST})$ is positive, whereas it has phase-lag characteristics when $\mathrm{Re}(\varepsilon_{ITO}+\varepsilon_{GST})$ is negative. According to Eq. (S3), the effective phase change of t_{GST} directly affects the propagation coefficient P_{IGI}, and eventually affecting the thin-film resonance wavelength. When the amorphous GST film is inserted, $\mathrm{Re}(\varepsilon_{ITO}+\varepsilon_{GST})$ is positive. Therefore, the phase-lead characteristics arise after passing through the GST film, which makes the effective cavity length of the IGI composite layer thicker, with such variance resulting in the red-shift of the resonance condition. On the other hand, during the crystalline GST insertion, the negative $\mathrm{Re}(\varepsilon_{ITO}+\varepsilon_{GST})$ leads to the blue-shift of the resonance. Using Eq. (S5), the enlargement of the resonance shift with the increase of d_{GST} (Fig. 3b of main text) can also be simply explained.

Part D. Viewing angle limit of our configuration

The theoretical limit of diffractive angle for 1um pixel pitch is about 15°. But the viewing angle of Fresnel hologram is also limited by distance between CGH panel and image plane. Let us show a brief supplementary schematic to clearly explain our condition,

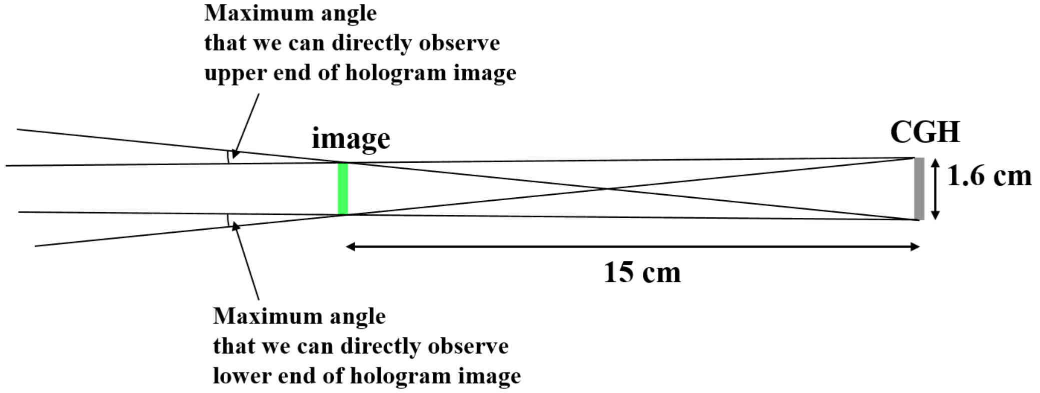

Figure S4. Schematic for explaining the viewing angle limitation in our hologram experiment.

Here, the relative scale of schematic is same as real length. The panel size is 1.6 cm, whereas distance between image and CGH is relative long (15 cm). Because the pixel pitch is 1um, minimum period is 2 um. Hence diffracted light from the panel can have theoretical maximum value of arcsin(532/2000) = -15~15°. However, only the maximum ray components of arcsin(1.6/15) ~= 6.1° can contribute to make a hologram image that we purposed. Therefore, the length between CGH panel and image restrict the viewing angle of

our system. Strictly speaking, the hologram image does not contain diffracted ray component larger than arcsin(1.6/15) ~= 6.1° in our configuration. If we make the hologram image close to the CGH pattern, viewing angle will be increased up to the theoretical diffraction limit. We think that it was our small mistake to design focal plane too far to perfectly show the best performance of our system, but it does not mean that our system cannot support large diffraction angle.

Part E. Effect of spacing between the pixels to diffraction efficiency

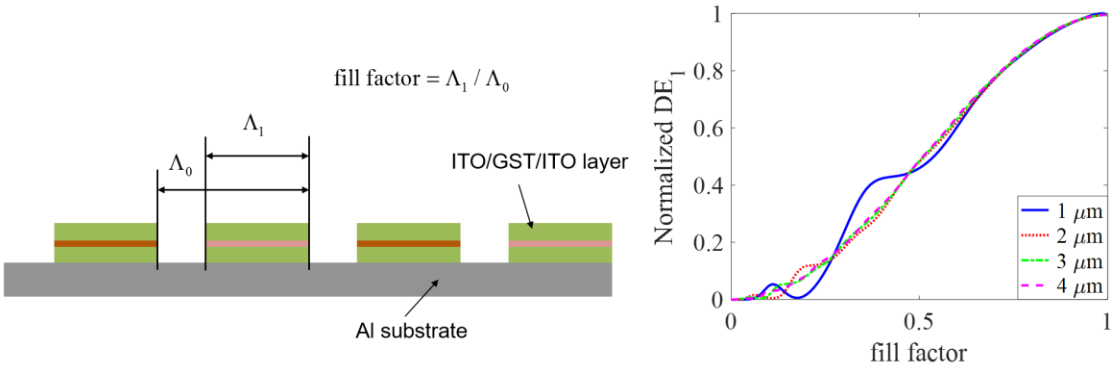

Figure S5. Effect of spacing between each pixels to their diffraction efficiency.

Although we checked for one-dimensional grating case, we tested how the portion of the active area affect to the diffraction efficiency by using the numerical simulations. According to the Fig. S5, here we define pixel fill factor as a portion of active pixel region compared to non-active region. It provide almost linear decreasing of diffraction efficiency until the fill factor is reduced to 0.5. Below this value, diffraction efficiency fluctuate according to the change of fill factor when pixel pitch is quite small such as 1 μm, but it still show nearly linear curve when pixel pitch is larger than 2 μm. The simulation shows the case of ITO-GST-ITO thickness of (30/7/30 nm), and the non-active region is assumed to air gap.

Part F. Reflection spectrum of the active region as used in the optoelectronic pixel modulation experiment

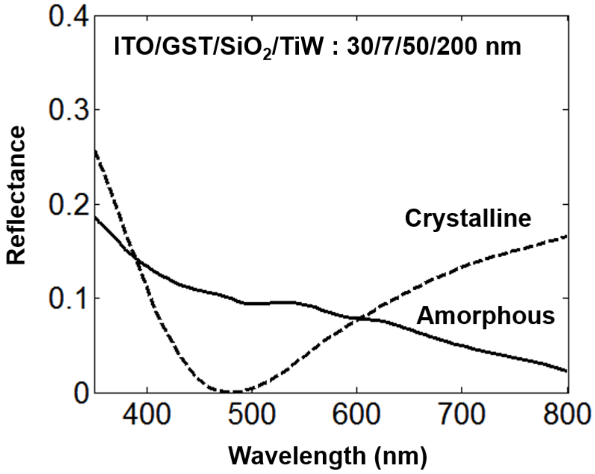

Figure S6. Reflectance of the ITO/GST/SiO_2/TiW layer before and after GST crystallization.

Part G. Electric pulse experiment for different thickness and active region size

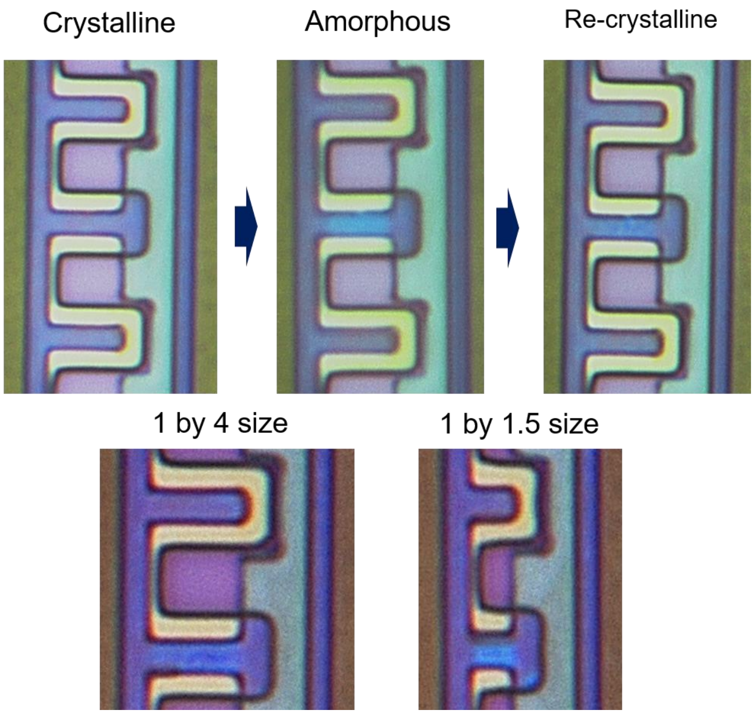

Figure S6. Figures show the electric pulse experiment for different thickness of ITO/GST/SiO2 = 30/7/220. This condition is nearly matched to the red-diffractive condition therefore the color of active

region shows blueish colors. Lower two figures show the phase change characteristics of 1 by 4 μm and 1 by 1.5 μm samples, respectively. The color difference between upper and lower images originates from different contrast settings in microscope.

An additional experiment sample for red-color diffractive panel is shown. Although the thickness characteristic is different (ITO/GST/SiO2 = 30/7/220, Note that color-sensitive condition is not unique because higher order FP resonance can also be used), we can clearly see blueish active regions before and after the GST phase transition. These two colors are nearly same as the colors shown in the red-selective CGH pattern in Fig. 4c. We tried to fix the thickness of upper ITO region in designing the dielectric layer to 30 nm, because driving voltage and current can be significantly changed when thickness of upper ITO is changed (device resistance may be changed). This is the reason that we do not choose 105/7/105 nm, but 30/7/220 nm for our new experiment results. The ITO thickness variation may affect to the driving voltage and current in unit pixel modulation, but designing the thickness of dielectric layers may have some degree of freedom so that the color-selective diffractive panel can also be fabricated without significantly changing the electric resistance of the unit pixel structure. In this experiment, reset pulse duration, relaxation time, and set pulse duration of 1 us, 1 us, and 10 us are applied, respectively. Therefore, about 1us+1us+10us = 12 us is needed for one cycle of phase transition in our experiment with current status. This value is still longer than state-of-art PRAM applications yet, but we assure that we can reduce phase transition time shorter in the future work.

As shown in the lower part of the Figure, our 1 μm x 1.5 μm pixel can also show color-changing characteristic by applying electric signal. But we find some unwanted color-changed region near the edge of TiW pattern. The reason of such unwanted color-changed region near the edge is that the edge resistance of the device is higher (or similar scale) than the narrow active region. Therefore we needed high aspect ratio of active region. We are trying to develop a new fabrication process to reduce the edge resistance. Well-known fabrication techniques such as spin-on-glass or nitride spacers can be considered to reduce the edge resistance.
